# Quality of Life and Functioning of People With Mental Disorders Who Underwent Deinstitutionalization Using Assisted Living Facilities: A Cross-Sectional Study

**DOI:** 10.3389/fpsyg.2021.622973

**Published:** 2021-05-25

**Authors:** Rejane Coan Ferretti Mayer, Maíra Ramos Alves, Sueli Miyuki Yamauti, Marcus Tolentino Silva, Luciane Cruz Lopes

**Affiliations:** Graduate Program in Pharmaceutical Sciences, University of Sorocaba, Sorocaba, Brazil

**Keywords:** mental health, quality of life, disability evaluation, deinstitutionalization, assisted living facilities

## Abstract

**Context:**

People with mental disorders can acquire long-term disabilities, which could impair their functioning and quality of life (QoL), requiring permanent care and social support. Systematic data on QoL and functioning, which could support a better management of these people, were not available.

**Objective:**

To analyze the QoL, level of functioning and their association with sociodemographic and clinical factors of people with mental disorders who underwent deinstitutionalization using assisted living facilities.

**Methods:**

A Cross-sectional study was conducted between July 2018 and July 2019, through interviews using the World Health Organization Quality of Life (WHOQOL-BREF) to determine the QoL scores, and the World Health Organization Disability Assessment Schedule (WHODAS 2.0) to determine the level of functioning. All adults (≥18 years old) with mental disorders, who underwent deinstitutionalization, users of assisted living facilities and assisted by the Psychosocial Assistance Centers III, in a city in the state of São Paulo, Brazil, were selected. For statistical analysis of the associated factors, Student’s *t*-test was used for dichotomous variables and ANOVA for polynomial variables. Pearson correlation coefficient was used to measure the association between QoL and functioning scores.

**Results:**

Out of 359 people who underwent deinstitutionalization with mental disorders, 147 met the eligibility criteria. The mean total score for the WHOQOL-BREF was 66.5 ± 13.4 and the mean score for WHODAS 2.0 was 10.4 ± 7.6. An association was found between people who were studying (*n* = 65.8; 95%CI, 63.5–68.1 *vs. n* = 73.9; 95%CI, 67.5–80.3; *p* = 0.04) and better WHOQOL-BREF QoL scores or WHODAS 2.0 levels of functioning (*n* = 10.9; 95%CI, 9.6–12.2 *vs. n* = 5.1; 95%CI, 2.5–7.7; *p* = 0.01). A weak negative correlation (*r* = 0.41) emerged between higher QoL scores and functioning improvement.

**Conclusion:**

This study indicates that the QoL of the sample is associated by their functioning levels, which, in turn, may reflect on their social interactions. Public policies that favor interventions increasing socialization of this population can result in better health outcomes. The QoL and functioning scores provide valuable insights to develop public policies more suited to this population profile.

## Introduction

Mental disorders can be defined as a group of signs and symptoms that clinically affect physical, psychological, behavioral, and cognitive aspects of a person (American Psychiatric Association, 2014). In some cases, they can cause long term incapacities, leading to impairments in people’s functioning and quality of life (QoL) and are responsible for 34% of the existing disabilities in the Americas (Hiany et al., 2018; Pan American Health Organization, 2018). People with severe mental disorders may need permanent care and social support in the community (World Health Organization and the Gulbenkian Global Mental Health Platform, 2014).

Deinstitutionalization movements first started in developed countries (Thornicroft and Bebbington, 1989; Yohanna, 2013; Hudson, 2019). In the United States, the decrease in hospital beds and changes toward community-based mental health services started in the 1950’s and a similar thing has happened with the United Kingdom (Thornicroft and Bebbington, 1989; Hudson, 2019). In Italy, this process took place in the 1960’s with a special focus on the experiences of Franco Basaglia, a pioneer in the anti-asylum movements in Italy, which had a significant impact on the deinstitutionalization movements in Brazil (Thornicroft and Bebbington, 1989; Amarante and Nunes, 2018). Since the deinstitutionalization process, mental health policies around the world passed through reformulations to provide care to the people who underwent deinstitutionalization recently (Shen and Snowden, 2014).

The Brazilian process diverged from these other countries, both because of the period — started the anti-asylum movements in the 1970’s and consolidated the deinstitutionalization laws in the 2000’s — and because of its structure. The mental health policy, introduced by the Psychiatric Reform Law (Brasil, 2001), is structured based on the humanization principles of the Brazilian Unified Health System and built in the form of a network (Ministério da Saúde, 2005, 2014; Santos et al., 2015; Sampaio and Bispo Júnior, 2021). It upheld the care in freedom and the reestablishment of the citizenship of people with mental disorders who underwent deinstitutionalization and were guided by the creation and maintenance, by the State, of services that substituted the asylum model, such as the Psychosocial Assistance Centers (in Portuguese, Centros de Atenção Psicossocial - CAPS) and assisted living facilities (Passos et al., 2013; Trape and Campos, 2017).

The assisted living facilities^1^ are dwellings, located in urban centers, aimed at sheltering people with mental disorders that, after a long period of hospitalization, have passed through the process of deinstitutionalization and lack familiar or community support. These services play an important role in retaking citizenship and reinserting the user into society, as it is through the symbolic and material appropriation of the dwelling and its surroundings that the rehabilitation process begins (Argiles et al., 2013; Cortes and Barros, 2017). Clinical care and individualized treatment plans are developed and applied at CAPS (Ministério da Saúde, 2004).

QoL comprises the individual’s relationships and perceptions, influenced by cultural determinants in relation to personal and social values under which the person lives (The WHOQOL Group, 1996). In addition, QoL encompasses important aspects of resocialization, environmental adaptation, and care for the individual (Costa et al., 2014; Passos and Portugal, 2015; Silva and Rosa, 2015).

The functioning of these people is also an important monitoring indicator. Functioning is understood as the bodily functions, the activities performed and the person’s participation, in relationship with environmental factors (barriers and enablers) (World Health Organization, 2007). In contrast, disabilities refer to impairments, limitations and restrictions to activities and participation, in relationship with the environment.

The functioning assessment and limitations of the person with mental disorders is important to identify the consequences related to the disorder, contributing to the choice of more effective interventions and priority areas for public resource allocation (Silveira et al., 2013).

QoL and functioning can be used as important parameters in the development of mental healthcare strategies, health condition monitoring and social reinsertion of these people, supporting the new assisted living facility implementations and improvement of existent ones.

This study analyzed the QoL, functioning and their relationship with sociodemographic and clinical aspects of people with mental disorder who underwent deinstitutionalization using the assisted living facilities in Brazil. In addition, the current study highlights the uniqueness of the Brazilian process, offering quantitative data about this population.

## Methods

### Study Design

A cross-sectional study was conducted between July 2018 and July 2019, through interviews using the World Health Organization Quality of Life instrument (WHOQOL-BREF) (The WHOQOL Group, 1996) to determine QoL scores and the World Health Organization Disability Assessment Schedule (WHODAS 2.0 – 12 items) to assess the levels of functioning (World Health Organization, 2010).

This study employed the Strengthening the Reporting of Observational Studies in Epidemiology (STROBE) checklist (Appendix 1) for the more precise and complete description of observational studies (Vandenbroucke et al., 2007).

### Context

The study was conducted in the city of Sorocaba, state of São Paulo, Brazil, in the CAPS III and the assisted living facilities.

Sorocaba is an important city in Brazilian mental health, because of the Vera Cruz Psychiatric Hospital, which, after years of operation, became a deinstitutionalization pole by the end of its activities (Prefeitura de Sorocaba, 2019). The city’s mental health plan, organized by the State Health Secretariat encompasses ten CAPS in total, one of each type for each Health Regional of the city. There are three CAPS of the type III. These offer specialized care to people with mental disorders and work with multidisciplinary teams including psychiatrists, nurses, nursing technicians, occupational therapists, psychologists, and social workers. They operate 24 h, having beds for nocturnal shelter and capacity to assist more than 1,000 people per month (Ministério da Saúde, 2014; Blog da Saúde, 2014).

There are 40 assisted living facilities in the city, with a capacity of ten, where people who underwent deinstitutionalization could return to live in the society, acquire responsibility, freedom, and autonomy over their daily routines, taking care of domestic activities and sharing houses with other people. Some facilities can have a caregiver, depending on the need and severity of the disorder of the residents.

### Participants

The participants were extracted from a population of 359 people who underwent deinstitutionalization living in assisted living facilities at Sorocaba city and assisted by the CAPS III unities, from July 2018 to July 2019. 114 failed to sign the informed consent form, leading to the availability of 245 people who were willing to participate in the research.

### Eligibility Criteria

Participants were selected if they underwent deinstitutionalization, were older than 18 years of age, users of assisted living facilities, assisted by the CAPS III in Sorocaba and had signed the informed consent form.

Participants were excluded if they failed to finish or refused to respond the questionnaires.

### Recruitment of Participants

The instruments were applied in the assisted living facilities, by two psychologists, RM and MA, with previous scheduling, both in the same day. The residents were reunited in their living room and invited to participate, in the presence of their trusted person, usually the caregiver or the technical reference of the house.

### Variables and Data Sources

In addition to the interviews, other complementary information was obtained from the medical records.

From the medical records, sociodemographic data (gender; age; marital status; education; financial autonomy; family ties) and clinical data (psychiatric hospital of origin; current diagnosis; diagnosis from hospitalization; comorbidities; time of hospitalization; time of deinstitutionalization; number of hospitalizations in psychiatric beds, CAPS beds and general hospital beds after deinstitutionalization; drug therapy) were extracted.

In the absence of any of this information above within the medical record, the health team was consulted. The team and the participant validated the missing or incomplete information in the medical record, to reduce the memory bias in this type of research design.

WHOQOL-BREF and WHODAS 2.0-12 items were applied both in the same day and only once to the participants.

WHOQOL-BREF (The WHOQOL Group, 1996) is a QoL questionnaire developed by the World Health Organization, composed of 24 items clustered into four domains: Physical health, Psychological, Social relationships, and Environment; and two items referring to the Self-evaluation of the QoL, totaling 26 items (Fleck et al., 2000; Pedroso et al., 2010). It was developed by specialists from different cultures as a generic intercultural instrument that could be used by different professionals (Angelim et al., 2015).

When compared to other widely used instruments, such as the 36-item Form Constructed to Survey Health Status (SF-36), EUROQOL (EQ-5D), Medical Outcomes Studies 36-item Short-Form (MOS SF-36) and Medical Outcomes Studies 12-item Short-Form (MOS SF-12), WHOQOL-BREF stands out for presenting the Social relationships and Environment domains, in addition to Physical health and Psychological domains (Almeida-Brasil et al., 2017; Skevington and Epton, 2018). This makes it possible to assess issues related to the person’s subjective perception over its contexts, distinctly from other instruments, which evaluate issues related to the functioning consequences of a given health condition (Almeida-Brasil et al., 2017).

These characteristics make WHOQOL-BREF a broader instrument about QoL perception and can be used in different knowledge areas (Castro et al., 2014; Skevington and Epton, 2018). It presents both low user bounce and data loss rates which makes it a more precise instrument (Skevington and Epton, 2018).

The WHODAS 2.0-12 items were used to assess the functioning outcome variable. The full version WHODAS 2.0 instrument has 36 items and is scored on a scale of 0-40, with higher scores indicating poor levels of functioning. It evaluates the functioning levels in six domains: Cognition, Mobility, Self-care, Getting along, Life Activities and Participation, which composes the domains of the International Classification of Functioning (World Health Organization, 2007). The 12-item instrument is a shortened version for assessments limited in time or because of other reasons that can cause an impediment to the 36-item instrument use (Ćwirlej-Sozańska et al., 2020). The WHODAS 2.0 - 12 items version used in the current study limits the calculation of scores by domains and scores range on a scale of 12-60 (World Health Organization, 2010).

Validation studies of the WHODAS 2.0 – 12 items showed that the instrument displays reliability to score functioning and health conditions, especially when applied to populations with mental disorders. In addition, because of its quick and easy application, its use has been recommended with this population (Carlozzi et al., 2015; Axelsson et al., 2017; Ćwirlej-Sozańska et al., 2020). Non-parametric analysis shows that the 12-item instrument works well within the different levels of disablement and it presented no differences when applied to both genders (Luciano et al., 2010). It has also been adapted and validated for the Brazilian context (Silveira et al., 2013).

### Biases, Confounding Variables, and Effect Modifiers

To avoid information bias and systematic errors related to data collection, high quality validated instruments were used. The researchers that collected data were trained to apply the instruments, following the orientations within each manual, to guarantee a standardized application, also avoiding observation, instrument assessment, and verification biases.

Possible recall bias within the data collection through interviews, due to the influence of the disorder severity and memory problems resulting from the temporal distance in which the questioned event occurred (the questionnaires asked about events occurred 2 weeks prior to its application), was reduced confronting the data with the person’s care team.

### Data Analysis

The quantitative data consisted of sociodemographic data and outcome variables, which were tabulated and compared. The scores obtained for each instrument, WHOQOL-BREF and WHODAS 2.0 – 12 items, were calculated following each respective manual instruction (The WHOQOL Group, 1996; World Health Organization, 2010).

For the WHOQOL-BREF scores, first a simple mean was calculated for the Likert-scored answers. Then, they were transformed into a score of 4-20 points and later transformed again into a scale of 0-100 points, in which scores closer to 100 indicate better QoL (The WHOQOL Group, 1996).

For WHODAS 2.0 – 12 items, we used the simple scoring method described in the manual. In this type of score, recommended for the 12 items version, the values assigned to each answer are summed, wherein “none” = 1, “mild” = 2, “moderate” = 3, “severe” = 4 and “extreme” = 5 (World Health Organization, 2010).

Statistical descriptive analysis was performed for the studied variables measured. Frequency was calculated for the categorical variables and means and standard deviations for the continuous variables. The association with the sociodemographic and clinical factors was assessed using the student’s t-test for the dichotomous variables and analysis of variance (ANOVA) for polynomial variables, using STATA (14.2 version) statistical program. The 95% confidence interval was standardized, and the level of significance was set at 5%.

A dispersion graph was plotted to show the correlation between QoL and functioning, that was assessed by Pearson correlation coefficient.

## Results

**FIGURE 1:**
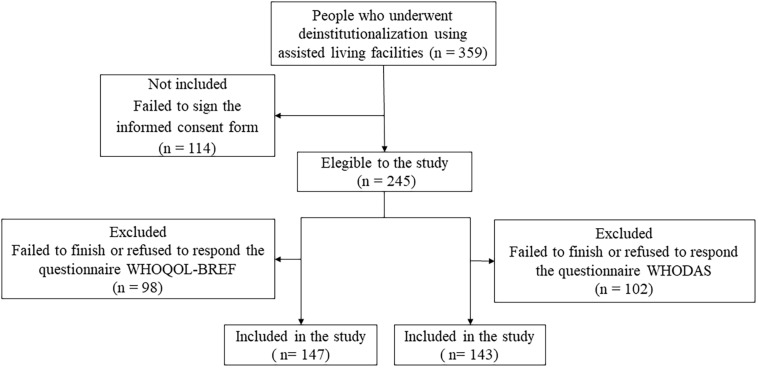
Flowchart of Sample Composition. WHOQOL-BREF, World Health Organization Quality of Life. WHODAS 2.0, World Health Organization Disability Assessment Schedule.

Out of 359 people who underwent deinstitutionalization living in assisted living facilities at Sorocaba city, 114 failed to sign the informed consent form, resulting in 245 people who were willing to participate in the research. During the WHOQOL-BREF application, 98 failed to finish or refused to respond the questionnaire and were excluded, resulting in 147 people who had completed the QoL assessment. In the WHODAS 2.0 – 12 items application, 102 failed to finish or refused to respond the questionnaire and were excluded, resulting in 143 people who had completed the functioning assessment (Figure 1).

About the population’s sociodemographic characteristics: the participants (*n* = 147) displayed a mean age of 51.5 ± 10.6 years, were predominantly male (*n* = 83, 56.5%), single (*n* = 136, 92.5%), not literate (*n* = 98, 66.7%), without paid work (*n* = 145, 98.6%), and beneficiaries of social programs (*n* = 135, 91.8%) (Table 1).

**TABLE 1:** Sociodemographic characteristics of people with mental disorders who underwent deinstitutionalization using assisted living facilities, Sorocaba city, 2019.

Variable	Full sample
Total, *n* (%)	147 (100.0)
**Sociodemographic characteristics**	
***Gender, n (%)***	
Male	83 (56.5)
Female	64 (43.5)
*Age in years (mean ± SD)*	51.5 ± 10.6
***Age, n (%)***	
≤40 years	18 (12.2)
41-50 years	55 (37.4)
51-60 years	47 (32.0)
>60 years	27 (18.4)
***Marital status, n (%)***	
Divorced	4 (2.7)
Separated	4 (2.7)
Single	136 (92.5)
Widow(er)	3 (2.0)
***Education, n (%)***	
Literate	49 (33.3)
Not literate	98 (66.7)
***Currently studying, n (%)***	
Yes	13 (8.8)
No	134 (91.2)
**Financial autonomy**	
Guardianship*, n (%)	
Wards	68 (46.3)
Without guardianship	79 (53.7)
***Benefit, n (%)***	
Yes	135 (91.8)
No	12 (8.2)
***Manages own benefit, n (%)***	
Yes	26 (17.7)
No	121 (82.3)
***Paid work, n (%)***	
Yes	2 (1.4)
No	145 (98.6)
***Family tie, n (%)***	
Yes	53 (36.1)
No	57 (38.8)
Not informed**	37 (25.2)

*Protection mechanism for those who, even in legal age, are unable to govern their own lives and to self-determine in wealth, due to disability, being this responsibility imposed to a natural person (Brasil, 2015).

**Data were not found in medical records and/or the health team was unable to inform.

*SD, Standard deviation.*

Schizophrenia spectrum disorders (*n* = 63, 42.9%) were the most prevalent diagnosis of mental disorders, followed by intellectual disabilities (*n* = 41, 27.9%). The mean time of hospitalization in psychiatric hospitals was 16.9 ± 8.8 years. Most of them were not admitted to a psychiatric bed (*n* = 143, 97.2%) and/or CAPS bed (*n* = 136, 92.5%) after deinstitutionalization (Table 2).

**TABLE 2:** Clinical characteristics of people with mental disorders who underwent deinstitutionalization using assisted living facilities, Sorocaba city, 2019.

Variable	Full sample
Total, *n* (%)	147 (100.0)
**Clinical characteristics**	
*Time of hospitalization in years (mean ± SD)*	*16.9 ± 8.8*
***Time of hospitalization, n (%)***	
≤10 years	33 (22.4)
11- 20 years	63 (42.9)
21 - 30 years	30 (20.4)
31 - 40 years	4 (2.7)
41 - 50 years	4 (2.7)
Not informed*	13 (8.8)
*Time of deinstitutionalization in years (mean ± SD)*	*3.5 ± 3.1*
***Time of deinstitutionalization, n (%)***	
≤5 years	126 (85.7)
6 - 10 years	13 (8.8)
11 - 15 years	6 (4.1)
16 - 20 years	1 (0.7)
Not informed*	1 (0.7)
**Hospitalizations (Admission or Readmission)**	
***Psychiatric bed, n (%)***	
No	143 (97.3)
Yes	4 (2.7)
***General hospital bed, n (%)***	
No	146 (99.3)
Yes	1 (0.7)
***Psychosocial assistance center bed, n (%)***	
No	136 (92.5)
Yes	11 (7.5)
***Current diagnosis, n (%)***	
Schizophrenia spectrum disorders (ICD 10**: F20 a F29)	63 (42.9)
Intellectual disabilities (ICD 10**: F70 a F79)	41 (27.9)
Schizophrenia spectrum disorders and Intellectual disabilities	25 (17.0)
Others	12 (8.2)
Not informed*	6 (4.1)
***Current clinical comorbidities, n (%)***	
Diabetes	6 (17.6)
Hypertensive diseases	8 (23.5)
Epilepsy	6 (17.6)
Diabetes and Hypertensive diseases	8 (23.5)
Diabetes, Hypertensive diseases and Metabolic disorder	1 (2.9)
Others	5 (14.7)
Without comorbidity	113 (76.9)
***Physical Disabilities, n (%)***	
No	139 (96.6)
Yes	8 (5.4)
**Drug Therapy**	
Polypharmacy (3 or more drugs) ***, n (%)	
No	7 (4.8)
Yes	140 (95.2)
***Number of psychotropic drugs in use, n (%)***	
None	2 (1.4)
1 drug	8 (5.4)
2 drugs	14 (9.5)
3 drugs	31 (21.1)
4 drugs	42 (28.6)
5 or + drugs	50 (34.0)

*Data were not found in medical records and/or the health team was unable to inform.

**ICD-10, International Classification of Diseases and Related Health Problems (10th Revision) of World Health Organization (1994).

***Includes all the medicines currently taken by the residents.

*SD, Standard deviation.*

About the level of functioning variable, we have only measured the global functioning score for our sample, since the WHODAS 2.0 – 12 items do not allow the calculation of scores by domains. The mean functioning score obtained was 10.4 ± 7.6.

The mean total score for the WHOQOL-BREF questionnaire was 66.5 ± 13.4. Between the domains, the greater mean score was for the QoL Self-evaluation domain (69.8 ± 21.8) and the lowest for the Psychological domain (63.7 ± 19.0) (Table 3).

**TABLE 3:** Mean transformed scores (0-100 scale) of WHOQOL-BREF obtained by the application in people with mental disorders who underwent deinstitutionalization using assisted living facilities, Sorocaba city, 2019.

Domains	Mean ± SD	Minimum value	Maximum value	Amplitude	Number of participants with lower values*, n (%)
Physical health	66.3 ± 14.8	25	96.4	71.4	23 (15.6)
Psychological	63.7 ± 19.0	0	100	100	20 (13.6)
Social relationships	68.5 ± 18.9	0	100	100	16 (10.9)
Environment	66.4 ± 16.9	0	100	100	16 (10.9)
QoL self-evaluation	69.8 ± 21.8	0	100	100	16 (10.9)
Final score	66.5 ± 13.4	14.4	95.2	80.8	18 (12.2)

Higher values correspond to better quality of life scores. The maximum value allowed by the scale is 100.

*values <1 Standard Deviation (SD).

Statistically significant associated factors related to sociodemographic and clinical variables and QoL or levels of functioning were not found, except for the “currently studying” variable (Tables 4, 5).

**TABLE 4:** Distribution of people with mental disorders who underwent deinstitutionalization using assisted living facilities by sociodemographic and clinical characteristics according to WHOQOL-BREF, 2019, *n* = 147.

WHOQOL-BREF (*N* = 147)				

Variables	N	Mean	95% CI*	*p* value
***Gender***				
Female	64	65.2	61.6 - 68.8	0.31^1^
Male	83	67.5	64.8 - 70.2	
***Age***				
≤ 40 years	18	67	61.2 - 72.8	0.92^2^
41- 50 years	55	67.3	64.1 - 70.5	
51 - 60 years	47	66.1	61.6 - 70.5	
> 60 years	27	65.3	60.3 - 70.3	
***Education***				
Not literate	98	67.3	64.6 - 69.9	0.34^1^
Literate	49	65	61.0 - 69.1	
***Currently studying***				
No	134	65.8	63.5 - 68.1	0.04^1^
Yes	13	73.9	67.5 - 80.3	
***Paid work***				
No	145	66.6	64.4 - 68.8	0.67^1^
Yes	2	62.5	50.3 - 74.7	
***Manages own benefit***				
No	121	67.3	65.0 - 69.6	0.11^1^
Yes	26	62.7	56.4 - 68.9	
Family tie**				
No	57	68.5	65.9 - 71.2	0.08^1^
Yes	53	64	59.7 - 68.4	
Current diagnosis of mental disorder**				
Intellectual disability	41	68.5	64.6 - 72.3	0.63^2^
Schizophrenia	63	65.6	62.3 - 68.9	
Schizophrenia and Intellectual disability	25	65	59.1 - 70.9	
Other	12	64.5	56.7 - 72.2	
***Intellectual disability***				
No	75	65.4	62.4 - 68.4	0.43^1^
Yes	66	67.2	63.9 - 70.5	
***Schizophrenia***				
No	53	67.6	64.1 - 71.1	0.35^1^
Yes	88	65.4	62.5 - 68.3	
***Other diagnosis***				
No	129	66.4	64.1 - 68.7	0.63^1^
Yes	12	64.5	55.8 - 73.1	
***Comorbidity***				
No	113	66.4	64.1 - 68.8	0.92^1^
Yes	34	66.7	61.4 - 72.1	
Time of hospitalization**				
≤ 10 years	33	68.5	64.7 - 72.3	0.01^2^
11- 20 years	63	64.9	61.7 - 68.1	
21 - 30 years	30	72.1	68.3 - 76.0	
31 - 40 years	4	54.4	34.2 - 74.5	
41 - 50 years	4	59.4	40.0 - 78.7	
Time of deinstitutionalization**				
≤ 5 years	126	66.3	63.8 - 68.7	0.76^2^
6 - 10 years	13	68.7	62.8 - 73.6	
11 - 15 years	6	70.2	61.4 - 79.0	
16 - 20 years	1	57.2	-	
***Polypharmacy***				0.26^1^
No	7	60.9	40.5 - 81.4	
Yes	140	66.8	64.6 - 68.9	
***Physical disability***				0.84^1^
No	139	66.6	64.3 - 68.8	
Yes	8	65.6	58.9 - 72.3	

*CI, Confidence Interval.

**Variable has uninformed data, disregarded in statistical calculations.

*^1^Student’s *t*-test values.*

*^2^ANOVA analysis of variance values.*

**TABLE 5:** Distribution of people with mental disorders who underwent deinstitutionalization using assisted living facilities by sociodemographic and clinical characteristics according to WHODAS 2.0, 2019, *n* = 143.

WHODAS (*N* = 143)				

Variables	N	Mean	95% CI*	*p* value
***Gender***				
Female	60	10.6	8.8 - 12.4	0.75^1^
Male	83	10.2	8.4 - 11.9	
***Age***				
≤ 40 years	20	9.8	6.0 -13.5	0.49^2^
41- 50 years	53	10.4	8.6 -12.2	
51 - 60 years	45	10.3	7.9 -12.6	
> 60 years	25	10.9	7.6 -14.2	
***Education***				
Not literate	94	11.2	9.7 - 12.8	0.05^1^
Literate	49	8.7	6.6 - 10.7	
***Currently studying***				
No	130	10.9	9.6 - 12.2	0.01^1^
Yes	13	5.1	2.5 - 7.7	
***Paid work***				
No	141	10.4	9.2 - 11.7	0.36^1^
Yes	2	5.5	26.3 - 37.3	
***Manages own benefit***				
No	116	10.4	9.0 - 11.8	0.87^1^
Yes	27	10.1	6.8 - 13.5	
Family tie**				
No	54	9.7	8.1 - 11.3	0.28^1^
Yes	52	11.3	8.8 - 13.8	
Current diagnosis of mental disorder**				
Intellectual disability	40	11.4	8.6 - 14.1	0.53^2^
Schizophrenia	60	9.5	8.0 - 11.0	
Schizophrenia and Intellectual disability	25	11.7	7.8 - 15.6	
Other	12	9.9	7.1 - 12.8	
***Intellectual disability***				
No	72	9.6	8.2 - 10.9	0.14^1^
Yes	65	11.5	9.2 - 13.8	
***Schizophrenia***				
No	52	11	8.8 - 13.3	0.51^1^
Yes	85	10.2	8.6 - 11.7	
***Other diagnosis***				
No	125	10.5	9.2 - 11.9	0.79^1^
Yes	12	9.9	6.8 - 13.1	
***Comorbidity***				
No	110	10.2	8.8 - 11.6	0.68^1^
Yes	33	10.8	7.9 - 13.8	
Time of hospitalization**				
≤ 10 years	31	8.4	6.0 - 10.7	0.28^2^
11- 20 years	64	10.9	8.9 - 13.0	
21 - 30 years	26	9.8	7.9 - 11.7	
31 - 40 years	3	14	11.7 - 16.3	
41 - 50 years	4	15	2.8 - 27.2	
Time of deinstitutionalization**				
≤ 5 years	122	10.4	9.0 - 11.8	0.62^2^
6 - 10 years	13	11.3	7.9 - 14.7	
11 - 15 years	6	7.7	2.7 - 12.7	
16 - 20 years	-	-	-	
***Polypharmacy***				
No	7	12.4	2.2 - 22.6	0.46^1^
Yes	136	10.3	9.0 - 11.5	
***Physical disability***				
Yes	136	10.1	8.9 - 11.3	0.05^1^
No	7	15.9	4.9 - 26.8	

* CI, Confidence Interval.

** Variable has uninformed data, disregarded in statistical calculations.

*^1^Student’s t-test values.*

*^2^ANOVA analysis of variance values.*

**FIGURE 2:**
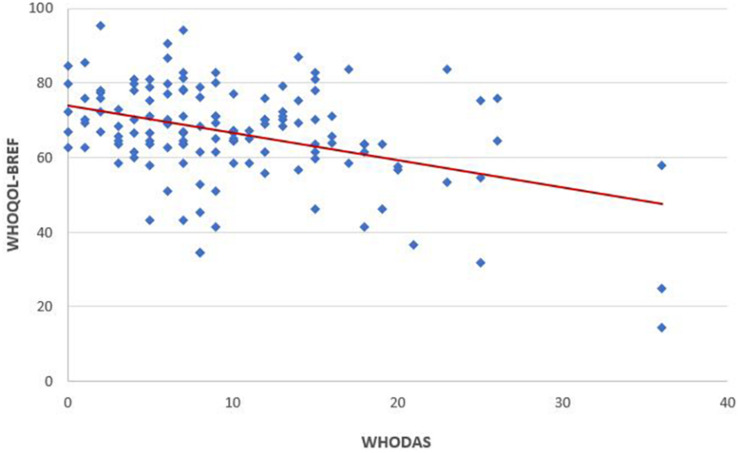
Correlation of quality of life (WHOQOL-BREF) to levels of functioning (WHODAS). Pearson correlation analysis shows a weak negative correlation of WHODAS versus WHOQOL-BREF (Pearson *r* = 0.41; *n* = 135). Line represents linear regression of data (*y* = –0.74x + 73.94; *r*^2^ = 0.1651).

A weak negative linear correlation emerged (r = 0.4) between the QoL and functioning scores such that the higher the decrease in the level of functioning, the lower the QoL scores (Figure 2).

## Discussion

### Main Findings

The study results indicated that in people with mental disorders who underwent deinstitutionalization using the assisted living facilities in Sorocaba, Brazil, the prevalent characteristics include middle-aged men, single, not literate, unemployed, without family ties or children, mainly with schizophrenia spectrum disorder or intellectual disabilities with a history of long years of hospitalization and a short time of deinstitutionalization. There seems to be an association between people who were currently studying, education and better levels of functioning and QoL scores. In addition, an association between physical disabilities and worst levels of functioning was observed. Furthermore, a weak negative linear correlation between QoL and functioning emerged. Other variables were not associated with the improvement of QoL or level of functioning.

### Comparison With Previous Studies

A few studies that used the WHOQOL-BREF instrument in people with schizophrenia obtained QoL mean scores higher than 80 (Mas-Exposito et al., 2011; Mohandoss, 2017), 20 points higher than the obtained by our sample. The QoL of the general population can be affected by factors related to gender, age, family ties, employment, social interaction, health conditions, among other factors (Gomez et al., 2017; Wang et al., 2017; Sum et al., 2018). Although these factors can influence the general population’s QoL, studies conducted in people with schizophrenia were not consistent regarding the existence of associated factors (Souza and Coutinho, 2006).

The prevalence of sociodemographic characteristics such as single middle-aged men, not literate, and unemployed, were also found in studies conducted in Rio de Janeiro (Alves et al., 2010), Piauí (Lago et al., 2014) and Pernambuco (França et al., 2017). However, these factors were not associated with lower QoL or functioning scores in our sample.

In addition to the mental disorder itself, which contributes to interpersonal difficulties, the isolation caused by a long hospitalization period reinforces social impairment and the need for reintegration, that could contribute to an improvement in QoL and functioning. These factors can explain, in part, the low score obtained in the Physical health and Psychological domains, since these domains cover issues about body health, energy, feelings, capacity perception, body perception and self-esteem (The WHOQOL Group, 1996; Pedroso et al., 2010).

The lack of employment, a prevalent characteristic in this sample, was also captured in previous studies (Alves et al., 2010; Lago et al., 2014; França et al., 2017) and could be related to stigma and prejudice, that is present in the employment sector, and in the society in general (Assunção et al., 2017). Our sample showed moderately low functioning mean values compared to other population studies (Ferrer et al., 2019). This can also partially explain the lack of employment.

To people with mental disorders, work can be therapeutic, an incentive to socialize and improve QoL, social recognition, and the development of abilities (Melo et al., 2015; Fernandes et al., 2017). A study conducted with 268 people with schizophrenia, using the WHOQOL-BREF, showed a positive association (*p* = 0.020) between being employed and better QoL scores (Pinho et al., 2018). Similarly, the associations found between people currently studying and better functioning and QoL scores show the importance of these sociodemographic aspects to elaborate intervention strategies that could develop abilities and functioning capacities of people with mental disorders. In addition, they might draw support from education and employment and, consequently, decrease their functioning disabilities (Picco et al., 2018).

However, although our sample has few family ties, the high mean scores obtained for the social relationship domain was surprising. A linear positive relationship between the support of the companions and friendships and an improvement in the QoL scores was observed (Portugal et al., 2016). One hypothesis to explain these findings is that, in assisted living facilities, the housemates become the new family, what ends up supplying the lack of fundamental support that can be offered by the family in the care (Costa et al., 2014; Dadalte et al., 2017).

It is worth mentioning that although the general QoL score was lower than the mean scores found in other populations (Cruz et al., 2011; Almeida-Brasil et al., 2017), the perception of QoL in this study, measured by the Self-evaluation domain of WHOQOL-BREF instrument, proved to be high. These high QoL perceptions scores could be explained by the fact that assisted living facilities are structured following the principles of humanized assistance of the Brazilian Unified Health System (Santos et al., 2015) and that, as stated by the World Health Organization (2001), the community-based mental health care, outside the psychiatric hospitals, have a positive effect in the clinical outcomes and QoL of people with chronic mental disorders, in addition to respecting human rights.

### Strengths and Limitations

This study presents original data about a vulnerable population that is directly affected by the care provided by the health system and by the severity of the mental disorder. The sociodemographic and clinical variables description used to verify the associated factors, represent important information to health professionals and local managers, to face the problem at this stage of the deinstitutionalization process. Data about QoL and functioning of people who underwent deinstitutionalization with mental disorders, users of assisted living facilities, since the Psychiatric Reform in Brazil (Brasil, 2001), were not available. These findings can contribute to better care management and amendment of public policies for this population.

Notwithstanding the fact that the sample can be considered epidemiologically small, it corresponds to the majority (∼70%) currently assisted by the assisted living facilities, in one of the cities in the region considered in the past, the largest asylum pole in the country. The prevalence of the diagnoses of complex severity and significant cognitive impairment helps to understand, in part, the profile of those previously institutionalized people, as well as the absence of bonds and family abandonment. In addition, this was a limiting factor for the person’s participation in the interviews about QoL and functioning since the cognitive impairment in about 40% of the people prevented them from understanding the questionnaire. Consequently, these people were excluded from this analysis. As a result, the data expressed here, although current on QoL and functioning, are not representative of a portion of the people who used assisted living facilities.

The design type of this study can be considered as a limitation for the result assessments since descriptive cross-sectional studies only show the prevalence of a given factor or characteristic, but do not establish cause-effect relationships, once the outcome and the exposure are assessed at the same time (Venancio, 2017).

## Conclusion

The QoL of people with mental disorders in this study was associated to their level of functioning, which in turn, can be reflected on the low employment rates and social interactions. Considering that this is a middle-aged population, that in a couple of years will become an elderly population, the mental health care should develop strategies that promote QoL and improve functioning of the people with mental disorders who use assisted living facilities, benefiting them and the society. Although the Brazilian mental health policy has been debatable, and the implementation process is incomplete, there is still room to rescue the financial, occupational, physical, and educational autonomy of the people with mental disorders, so that they can enjoy the social interaction benefits from which they were previously deprived.

The QoL and functioning scores in this study provide valuable data to develop public policies more suited to this population profile. Studies of national inquiry, comparing the QoL scores of these people, users of assisted living facilities, and those who returned to live with their families, and the type of care they receive in psychosocial attention networks could provide important information for interventions aimed at these people and better inform implementation of public policy.

## Data Availability Statement

The original contributions presented in the study are included in the article/Supplementary Material, further inquiries can be directed to the corresponding author/s.

## Ethics Statement

The study protocol was approved by the Brazilian Research Ethics Committee (protocol n° 2.600.954/2018) and by the Municipal Government of Sorocaba City (protocol n° 26022018). All subjects gave written informed consent in accordance with the Declaration of Helsinki.

## Author Contributions

LL and RM contributed to the study conception and design. MA and RM organized the database. MS and RM performed the statistical analysis. RM wrote the first draft of the manuscript. MA, SY, LL, and RM wrote sections of the manuscript. All authors contributed to the manuscript revisions, readings, and approved the submitted version.

## Conflict of Interest

The authors declare that the research was conducted in the absence of any commercial or financial relationships that could be construed as a potential conflict of interest.

## Footnotes

1 The assisted living facilities are described by the Medical Subject Headings (MeSH) as “A housing and health care alternative, combining […] housing, personalized supportive services and health care designed to meet the needs […] of those who need help with activities of daily living”. In Brazil, they are called ‘*Serviços Residenciais Terapêuticos*’, and work more as a form of social assistance than as a form of clinical assistance, this one offered by Psychosocial Assistance Centers.
